# Measurement of ice friction and aerodynamic drag for sliding on ice: Faster sliding in winter sports

**DOI:** 10.1016/j.mex.2022.101899

**Published:** 2022-11-05

**Authors:** Marina Cerpinska, Karlis Agris Gross, Janis Viba, Martins Irbe

**Affiliations:** Riga Technical University, Biomaterials Research Laboratory, Institute of Mechanics and Machine building, Latvia

**Keywords:** Skeleton, Sliding friction, Accelerometer, Stick and slip

## Abstract

This Method Article is co-submitted with the article Nr. JTRI_106967 in “Tribology International”. In the article we investigate the reasons behind a change in friction coefficient at low sliding velocities for a skeleton on ice. To complete the study a numerical model was created with air drag and the coefficient of friction in an equation representing sliding on ice. An experiment with two measurement systems, timing sensors on an ice track and a portable accelerometer at the base of the skeleton, was performed. A numerical model was amended with experimental data. Finally, changes of friction coefficient were analyzed, the difference in results provided by experiment and numerical calculations were illustrated, and conditions at the start when static friction transitioned to kinetic friction highlighted. The main findings about the methods used were as follows:

• Portable accelerometer helped to define the speed for transition to smooth sliding;

• The numerical model of time and velocity had greater error;

• The numerical model of distance and velocity was very close to experimental results.

## Graphical abstract

The abstract represents a method with the mostaccurate model according to experimental results.

**Figure:**
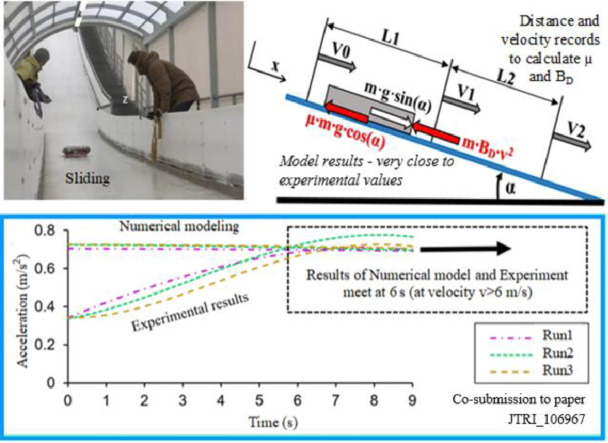

SPECIFICATIONS TABLE

**Table:** 

Subject Area	Materials Science
More specific subject area	Friction of steel-on-ice
Method name	Modeling of resistance forces for sliding
Name and reference of original method	L. Poirier, E. P. Lozowski, S. Maw, D. J. Stefanyshyn, R. I. Thompson (2011) Experimental analysis of ice friction in the sport of bobsleigh Sport. Eng. 14 67–72. DOI: 10.1007/s12283-011-0077-0
Resource availability	Available at: https://link.springer.com/article/10.1007/s12283-011-0077-0

The purpose of the investigation is to establish a method to assess sliding on ice, as feedback, for addressing different factors that affect the sliding speed. At the present stage, a handful of influencing factors are thought to influence the extent of sliding, but their relative influence remains undetermined. New method will provide a theoretical model for an analysis of a real-time sliding event. The best starting situation for using this analysis method is on a straight inclined ice track, and so is directly applicable to winter sports such as skeleton, luge and bobsled.

The experimental work has been conducted at the bobsled push-start facility in Sigulda, Latvia. Timing sensors at the side of the track register the body as it slides past. This concept can also be transferred to the ice track used in competitions to obtain reference data on the ice track. In this way, the sliding ability on ice can be assessed for those preparing to participate in a skeleton, luge or bobsled competition. Similarly to a weather forecast that provides “feels like” description that factors in the air temperature, humidity and wind, a “slipperiness” could be given for the ice upon which the sliding competition will occur. This information gives an indication of “sliding is like”, akin to “feels like” reports of the weather and will be useful for preparation of the sled for sliding, or understanding the gripping situation where safety on ice is important.

This model can be further applied to the bobsled track in straight sections, that only require the installation of timing sensors. Further developments could look at the use of this method by athletes. Athletes would be given a portable accelerometer that is fixed to the bottom of the skeleton for complementary information collected and analyzed from timing sensors. Development of this method could provide a real-time display on a screen for viewing by each athlete.

Two models were investigated to determine the most accurate match to sliding of a skeleton down a 50 m long ice track. One model will use time and velocity as input data, while the other model will look at distance and velocity as input data.

## Method details

Poirier et al. [1] suggested that the drag constant during deceleration of the sled can be introduced as *α*. The proposed air resistance coefficient is *B_D_*. The difference of our proposal - the mass is included into a new coefficient, because this step is handy for further integration, obtaining $B_{D}=\frac{\rho \cdot D\cdot A}{2\cdot m}$_._ Thus, the equation of sliding in differential equation form becomes (1):

(1) $$m\cdot \ddot{x}=m\cdot g\cdot \sin\left(\alpha \right)-\mu \cdot m\cdot g\cdot \cos\left(\alpha \right)-m\cdot B_{D}\cdot {\left(\dot{x}\right)}^{2}$$

where m is mass,$\;\ddot{x}$ is acceleration, μ is the steel-on-ice friction coefficient, $B_{D}$ is the air resistance coefficient,$\;{\left(\dot{x}\right)}^{2}$ is the velocity at a given moment; g is the gravitational constant and α is the incline angle.

### Recording time and velocity

The differential Eq. (1) was directly integrated over time, giving the relationship between velocity *v* and time *t* (2.1).

(2.1) $$t\left(v\right)-t_{0}=m\int_{v_{0}}^{v}\frac{dv^{\prime }}{F\left(v^{\prime }\right)}$$

where t(v) is a time at a given moment; v is a velocity at a given moment; $v_{0}$ and $t_{0}$ are the initial velocity and time; respectively $v^{\prime }$ is the parameter for integration.

Inserting $F(v^{\prime })$ from eq. $F\left(v^{\prime }\right)=m\cdot g\cdot \sin\left(\alpha \right)-\mu \cdot m\cdot g\cdot \cos\left(\alpha \right)-m\cdot B_{D}\cdot {\left(v^{\prime }\right)}^{2}$ into Eq. (2.1) provides Eq. (2.2):

(2.2) $$t\left(v\right)-t_{0}=\int_{v_{0}}^{v}\frac{dv^{\prime }}{g\cdot \sin\left(\alpha \right)-\mu \cdot g\cdot \cos\left(\alpha \right)-B_{D}\cdot v^{{}^{\prime }2}}$$

**Fig. 1:**
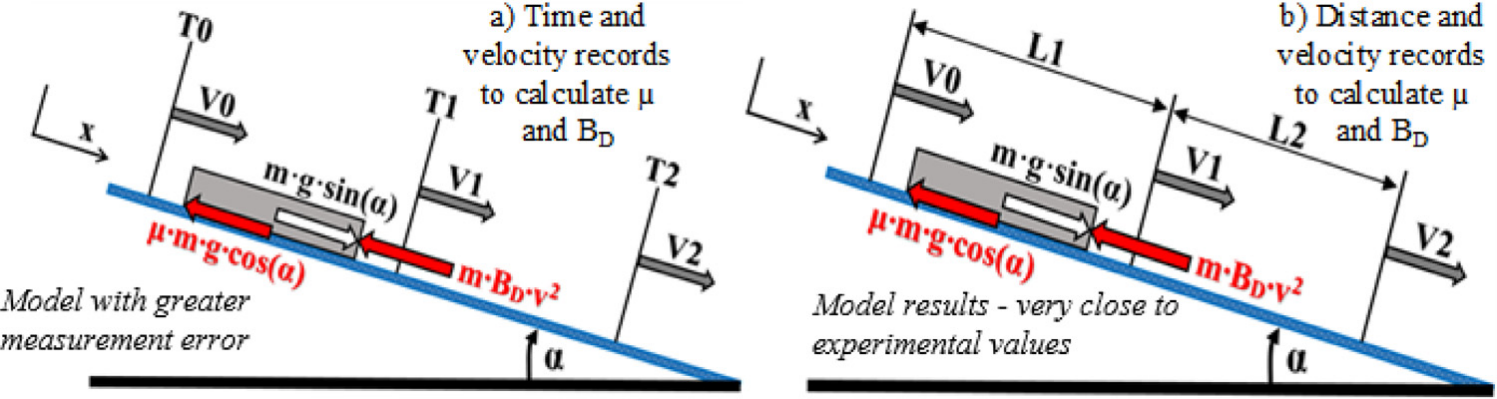
Model showing the forces acting on the sliding body and the three sets of recorded data: a) time and velocity; b) distance and velocity.

To solve Eq. (2.2) only time and velocity are required, as shown in Fig. 1 a). The time was measured using optical sensors. Velocity was calculated from the experiment by dividing the traveled distance by the actual time (measured with optical sensors) required to slide the respective distance.

**Fig. 2:**
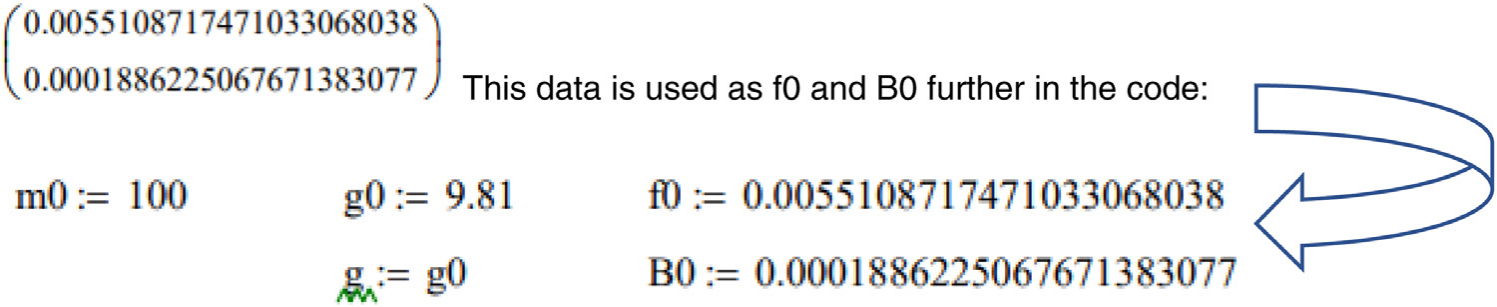
Experimental values for the numerical simulation in the code.

**Fig. 3:**
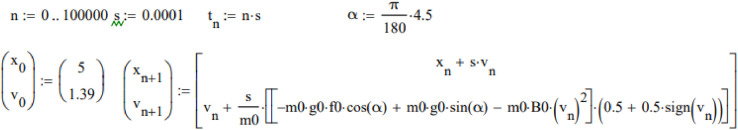
The explicit Euler method code.

### Recording distance and velocity

The differential Eq. (1) was directly integrated over displacement x, giving a relationship between velocity vand displacement x (3.1):

(3.1) $$x\left(v\right)-x_{0}=m\int_{v_{0}}^{v}\frac{v^{\prime }dv^{\prime }}{F\left(v^{\prime }\right)}$$

where *x(v)* is a coordinate at a given moment; *x_0_* is the coordinate at the start, *F(v’)* is a function defined in equation $F\left(v^{\prime }\right)=m\cdot g\cdot \sin\left(\alpha \right)-\mu \cdot m\cdot g\cdot \cos\left(\alpha \right)-m\cdot B_{D}\cdot {\left(v^{\prime }\right)}^{2}$.

Inserting *F(v’)* from eq. above into eq. (3.1) provides Eq. (3.2):

(3.2) $$x\left(v\right)-x_{0}=\int_{v_{0}}^{v}\frac{v^{\prime }\cdot dv^{\prime }}{g\cdot \sin\left(\alpha \right)-\mu \cdot g\cdot \cos\left(\alpha \right)-B_{D}\cdot v^{\prime 2}}$$

### Calculating coefficients with input data from instantaneous records

Time measurements at three locations on the ice track (as per Fig. 1), make it possible to calculate the resistance coefficients μ and $B_{D}$ (as shown in the paper, Results section – the table in Fig. 5).

The differential Eq. (3.2) is integrated over time in two stages, with time and velocity data from the experiment; *T0, V0* for the initial condition*, T1, V1* during sliding and *T2, V2* as the final condition (Fig. 1 a)). For integration, a constant *C_1_* is required. To calculate *C_1_*, motion at time *t_0_ =0 s* is analyzed. Inserting a zero value at *t_0_ =* T0 and $V_{0}$ gives an equation for calculating the first integration constant $C_{1}$ as shown in Eq. (4.0):

(4.0) $$-\frac{a\tan\left(\frac{\sqrt{B_{D}}\cdot V_{0}}{\sqrt{\mu \cdot g\cdot \cos\left(\alpha \right)-g\cdot \sin\left(\alpha \right)}}\right)}{\sqrt{B_{D}\cdot \mu \cdot g\cdot \cos\left(\alpha \right)-B_{D}\cdot g\cdot \sin\left(\alpha \right)}}=0+C_{1}$$

The constant *C_1_* is then used to form a relationship between motion at the end of the first stage of the sliding (4.1) and at the end of the second stage of sliding (4.2).

(4.1) $$\frac{a\tan\left(\frac{\sqrt{B_{D}}\cdot V_{1}}{\sqrt{\mu \cdot g\cdot \cos\left(\alpha \right)-g\cdot \sin\left(\alpha \right)}}\right)}{\sqrt{B_{D}\cdot \mu \cdot g\cdot \cos\left(\alpha \right)-B_{D}\cdot g\cdot \sin\left(\alpha \right)}}=-T_{1}+\frac{a\tan\left(\frac{\sqrt{B_{D}}\cdot V_{0}}{\sqrt{\mu \cdot g\cdot \cos\left(\alpha \right)-g\cdot \sin\left(\alpha \right)}}\right)}{\sqrt{B_{D}\cdot \mu \cdot g\cdot \cos\left(\alpha \right)-B_{D}\cdot g\cdot \sin\left(\alpha \right)}}$$

(4.2) $$\frac{a\tan\left(\frac{\sqrt{B_{D}}\cdot V_{2}}{\sqrt{\mu \cdot g\cdot \cos\left(\alpha \right)-g\cdot \sin\left(\alpha \right)}}\right)}{\sqrt{B_{D}\cdot \mu \cdot g\cdot \cos\left(\alpha \right)-B_{D}\cdot g\cdot \sin\left(\alpha \right)}}=-T_{2}+\frac{a\tan\left(\frac{\sqrt{B_{D}}\cdot V_{1}}{\sqrt{\mu \cdot g\cdot \cos\left(\alpha \right)-g\cdot \sin\left(\alpha \right)}}\right)}{\sqrt{B_{D}\cdot \mu \cdot g\cdot \cos\left(\alpha \right)-B_{D}\cdot g\cdot \sin\left(\alpha \right)}}$$

where *V_0_, V_1_* and *V_2_* are velocities at the start, at the 2nd location and at the end. $T_{1}$ and $T_{2}$ were 7.6 s and 1.2 s in this study. As a result, two Eqs. (4.1) and (4.2) with two unknowns (*μ* and *B_D_*) are obtained. For this study the calculation of coefficients *μ* and *B_D_* was done numerically using the MathCAD software Solve block "Given-Find".

### The model with lowest measurement error uses distance and velocity records

Similarly, the resulting differential Eq. (3.2) for the model presented in Fig. 1 b) can be used to determine the resistance force coefficients *μ* and *B_D_*. Then, the integration constant $C_{2}$ can be calculated using initial velocity $V_{0}$ and position $x_{0}=0$ providing Eq. (5.0):

(5.0) $$\frac{\ln\left(g\cdot \left(\sin\left(\alpha \right)-\mu \cdot \cos\left(\alpha \right)\right)-B_{D}\cdot v_{0}^{2}\right)}{2\cdot B_{D}}=0+C_{2}$$

The constant *C_2_* from the two sliding stages will provide the relationships at the end of the first stage (5.1) and at the end of the second stage (5.2):

(5.1) $$\frac{\ln\left(g\cdot \left(\sin\left(\alpha \right)-\mu \cdot \cos\left(\alpha \right)\right)-B_{D}\cdot v_{1}^{2}\right)}{2\cdot B_{D}}=-L_{1}+\frac{\ln\left(g\cdot \left(\sin\left(\alpha \right)-\mu \cdot \cos\left(\alpha \right)\right)-B_{D}\cdot v_{0}^{2}\right)}{2\cdot B_{D}}$$

(5.2) $$\frac{\ln\left(g\cdot \left(\sin\left(\alpha \right)-\mu \cdot \cos\left(\alpha \right)\right)-B_{D}\cdot v_{2}^{2}\right)}{2\cdot B_{D}}=-L_{2}+\frac{\ln\left(g\cdot \left(\sin\left(\alpha \right)-\mu \cdot \cos\left(\alpha \right)\right)-B_{D}\cdot v_{1}^{2}\right)}{2\cdot B_{D}}$$

where *L_1_* and *L_2_* are the distances of the first and second stages, specifically 35 m and 10 m in this study. As a result, two Eqs. (5.1) and (5.2) with two unknowns (*μ* and *B_D_*) are obtained, and unknown coefficients could be calculated. This is done in the code provided below using the Given-Find solve block.

### Method validation in MathCAD

For the code we use experimental values obtained from measurements of Run1 of the experiment described in preceding paper JTRI_106967, first row – friction coefficient f0, second row – air resistance coefficient B0:

The explicit Euler method of step integration is used to solve the problem numerically, the step size s was chosen to be 0.0001 for higher precision, the number of steps n was chosen to be 100000 to fit measurement time. One step of the Euler method for displacement $x_{n}$ and velocity $v_{n}$ is $x_{n+1}$ and $v_{n+1}$; $x_{0}$ and $v_{0}$ are guess values.

**Fig. 4:**
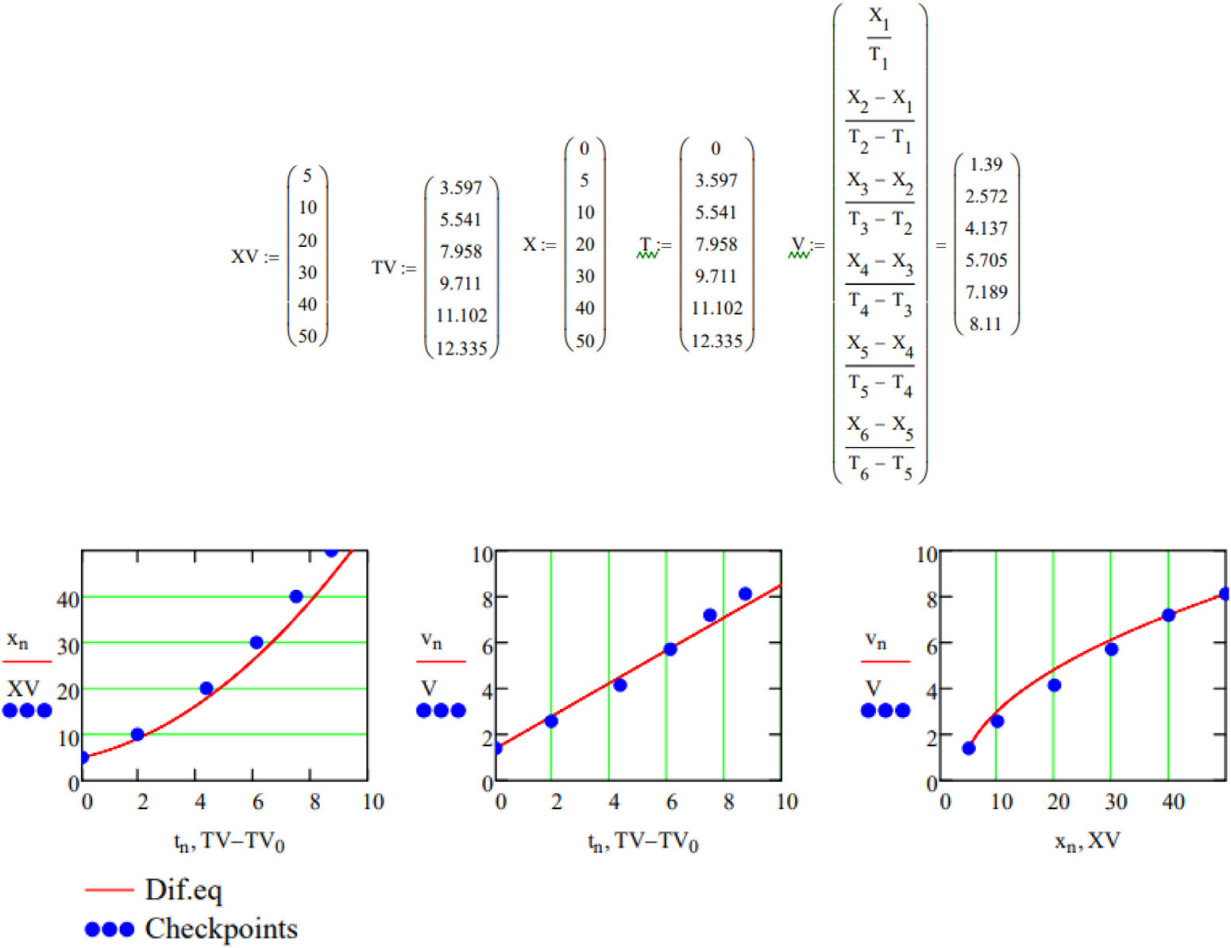
Comparison charts of numerical model (red line) and experimental data (blue dots)

The resultant lines of $x_{n}$and $v_{n}$ are plotted with red lines in the comparison charts in Fig. 4. Data from experiment, given below, is represented with blue points (XV – experimental checkpoint values of distance and V - experimental checkpoint values of velocity) in comparison charts in Fig. 4. To adjust values collected from experiment to start from zero, horizontal line of the plot for experimental values is modified to be TV-TV_0_.

### Calculating coefficients of friction and air resistance from instantaneous motion records

**Fig. 5:**
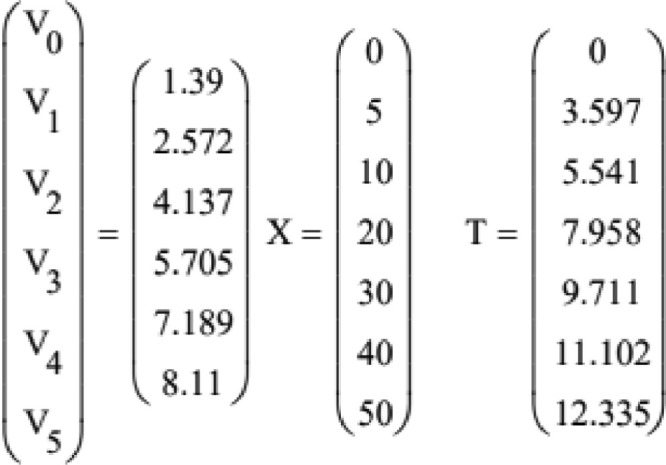
Instantaneous motion records from experiment

Motion records that were used to verify the model are provided in Fig. 5:

**Fig. 6:**
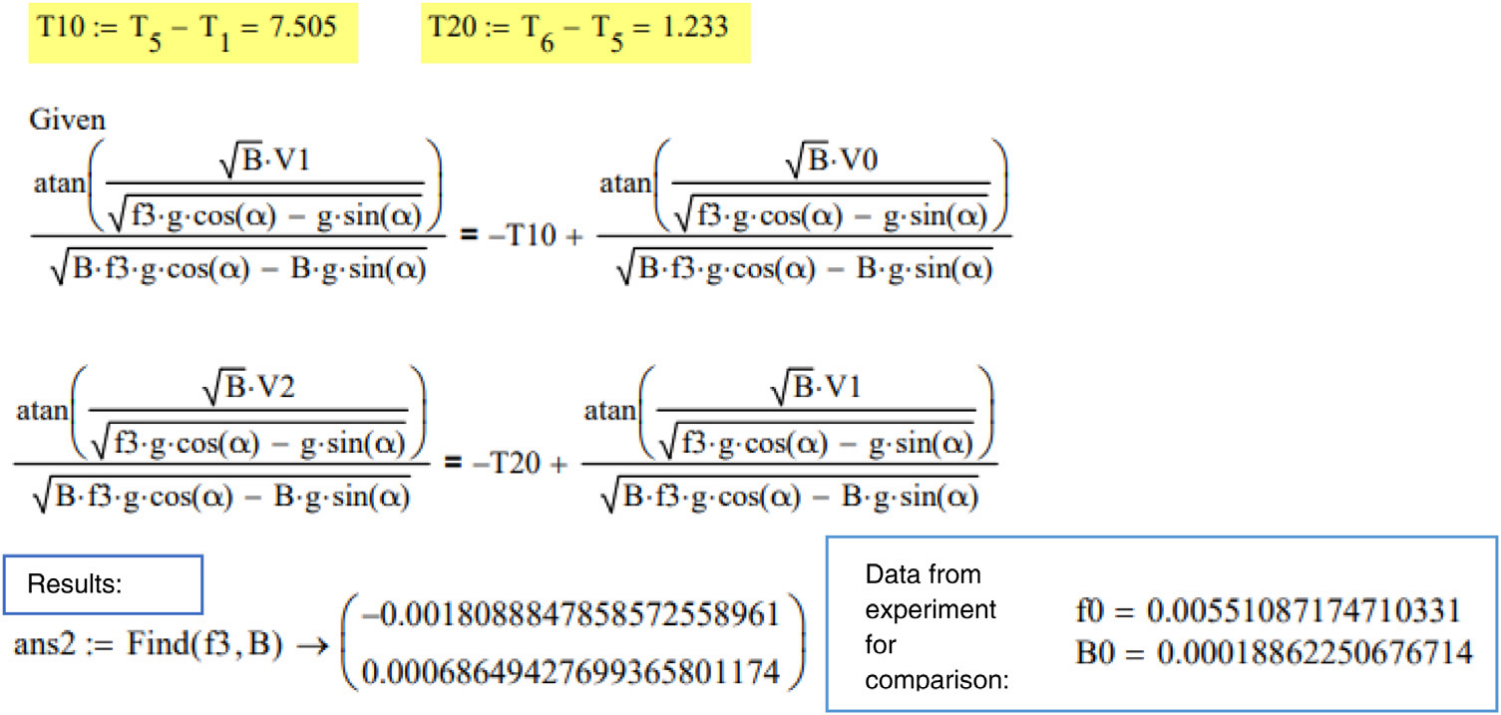
MathCAD code and results for Model A

**Fig. 7:**
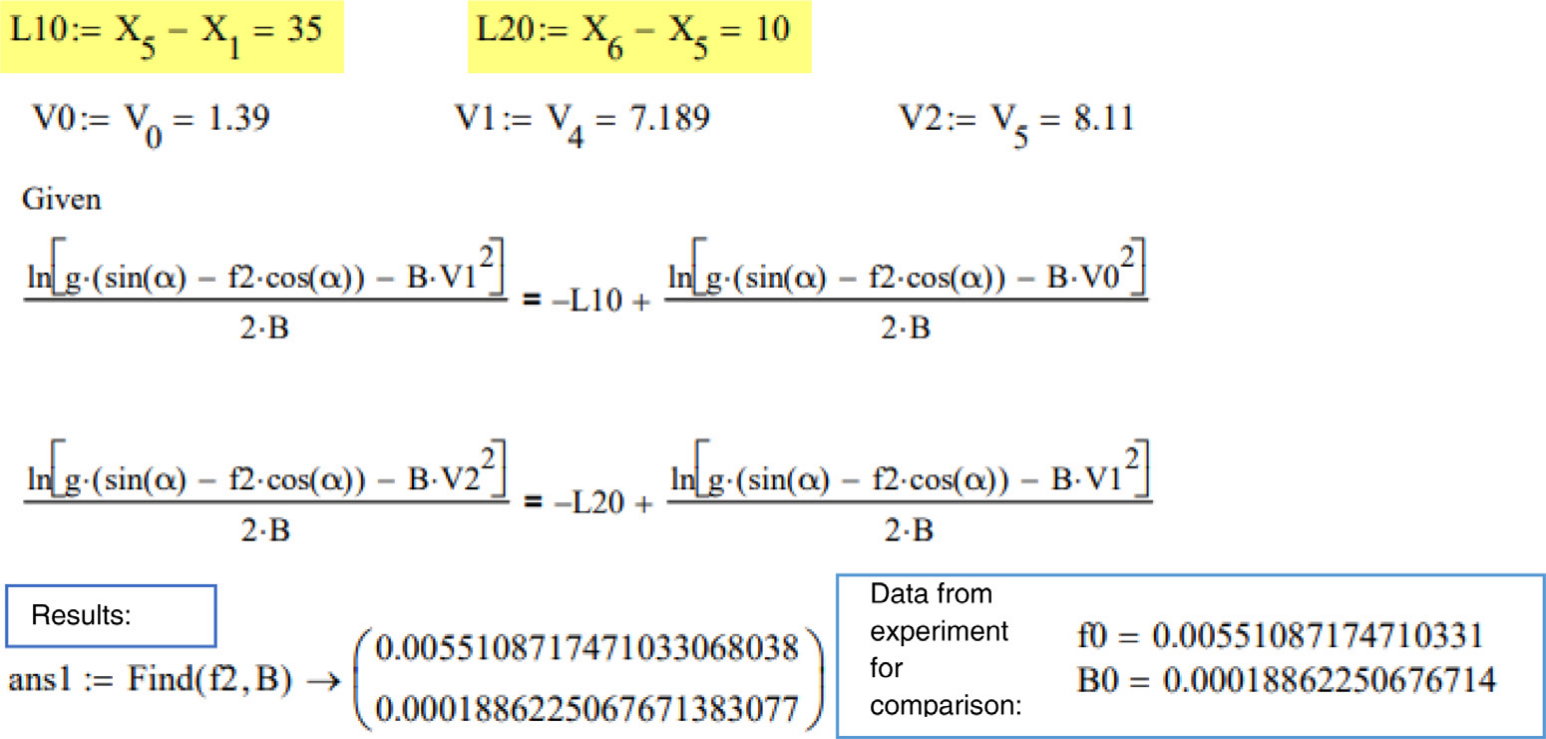
MathCAD code and results for Model B

## Model A. (Fig. 1 a) Time and velocity are known

The time in seconds when sliding object is passing by optical sensors is considered as known and is highlighted yellow in the code. Given-Find solve block is used for two equations with two unknowns to find ice friction and air resistance coefficients f3,B.

Conclusion for model A: The results obtained for model presented in Fig. 1.a (time and velocity known) are realistic, but they are not close to the experimental values. This is explained by practical reasons. While distance between sensors was measured very precisely, measured time when the skeleton passes the sensor consists of a measurement error.

## Model B. (Fig. 1 b) Distance and velocity are known

In this example, the distance in meters between optical sensors is known and is highlighted yellow in the code. Given-Find solve block for two equations with two unknowns is used to find ice friction and air resistance coefficients f2,B.

Conclusion for Model B: The results are very close to the experimentally obtained values for the method where distance and velocity are known (this corresponds to the mathematical model presented in Fig. 1.b). This method is presented in the paper and used to compare mathematical model data and experimental data.

## Declaration of Competing Interest

The authors declare that they have no known competing financial interests or personal relationships that could have appeared to influence the work reported in this paper.

Nothing to declare

Supplementary material *and/or* Additional information:

## Data Availability

Data will be made available on request.
